# Complexity of COVID-19 Dynamics

**DOI:** 10.3390/e24010050

**Published:** 2021-12-27

**Authors:** Bellie Sivakumar, Bhadran Deepthi

**Affiliations:** Department of Civil Engineering, Indian Institute of Technology Bombay, Powai, Mumbai 400076, India; 204040009@iitb.ac.in

**Keywords:** infectious diseases, coronavirus, COVID-19, nonlinear dynamics, chaos theory, phase space reconstruction, false nearest neighbor algorithm

## Abstract

With population explosion and globalization, the spread of infectious diseases has been a major concern. In 2019, a newly identified type of Coronavirus caused an outbreak of respiratory illness, popularly known as COVID-19, and became a pandemic. Although enormous efforts have been made to understand the spread of COVID-19, our knowledge of the COVID-19 dynamics still remains limited. The present study employs the concepts of chaos theory to examine the temporal dynamic complexity of COVID-19 around the world. The false nearest neighbor (FNN) method is applied to determine the dimensionality and, hence, the complexity of the COVID-19 dynamics. The methodology involves: (1) reconstruction of a single-variable COVID-19 time series in a multi-dimensional phase space to represent the underlying dynamics; and (2) identification of “false” neighbors in the reconstructed phase space and estimation of the dimension of the COVID-19 series. For implementation, COVID-19 data from 40 countries/regions around the world are studied. Two types of COVID-19 data are analyzed: (1) daily COVID-19 cases; and (2) daily COVID-19 deaths. The results for the 40 countries/regions indicate that: (1) the dynamics of COVID-19 cases exhibit low- to medium-level complexity, with dimensionality in the range 3 to 7; and (2) the dynamics of COVID-19 deaths exhibit complexity anywhere from low to high, with dimensionality ranging from 3 to 13. The results also suggest that the complexity of the dynamics of COVID-19 deaths is greater than or at least equal to that of the dynamics of COVID-19 cases for most (three-fourths) of the countries/regions. These results have important implications for modeling and predicting the spread of COVID-19 (and other infectious diseases), especially in the identification of the appropriate complexity of models.

## 1. Introduction

Infectious diseases are diseases caused by living organisms, such as viruses and bacteria. They can be passed from person to person. Some of the common infectious diseases include Hepatitis B, Malaria, Measles, Influenza, Chickenpox, Tuberculosis, Cholera, Typhoid, HIV/AIDS, Dengue, and Pneumonia. Infectious diseases are among the most dangerous health issues faced by humanity, since they can easily spread from person to person, over a large spatial extent, and over a long period of time. In this regard, population explosion and globalization have and continue to play a key role in the spread of infectious diseases. Globally, infectious diseases cause millions of deaths annually and also cost a huge amount of money for prevention and treatment. Therefore, an adequate understanding of the dynamics of infectious diseases is key to saving our lives and economy.

Coronavirus is a type of virus. Coronaviruses are named for their appearance. Under the microscope, the viruses look like they are covered with pointed structures that surround them like a corona, or crown. There are many different kinds of coronaviruses. Some of them cause colds or other mild respiratory (nose, throat, lung) illnesses. Other coronaviruses cause more serious diseases. During the twenty-first century, coronaviruses had earlier caused severe acute respiratory syndrome (SARS) and the Middle East respiratory syndrome (MERS). More recently, in 2019, a newly identified type of coronavirus caused an outbreak of respiratory illness, popularly known as COVID-19. The 2019 Coronavirus disease COVID-19 is caused by severe acute respiratory syndrome coronavirus 2 (SARS-CoV-2). The COVID-19 outbreak was identified in Wuhan, China, in December 2019 (WHO, Novel Coronavirus—China; see https://www.who.int/csr/don/12-january-2020-novel-coronavirus-china/en/ (accessed on 26 November 2021) [1]. The World Health Organization (WHO) declared the COVID-19 outbreak a Public Health Emergency of International Concern on 30 January 2020 and a pandemic on 11 March 2020. As of 11 December 2021, almost 269 million cases of COVID-19 have been reported in 223 countries, territories, and areas around the globe, resulting in almost 5.3 million deaths (World Health Organization, 2021; see https://www.who.int/emergencies/diseases/novel-coronavirus-2019 (accessed on 26 November 2021) [2]. The COVID-19 cases and deaths continue to grow in many parts around the world, including with new variants of COVID-19 over the past year (the variant Omicron, first identified in South Africa, being the latest). In addition to its obvious impacts on human health, COVID-19 also impacts numerous other sectors of our society, including the economy, education, environment, food production and distribution, tourism, migration and refugees, and social activities [3,4,5,6,7,8,9,10].

There is now a broad consensus that COVID-19 is spread through droplets released into the air when an infected person coughs or sneezes. The droplets generally do not travel more than a few feet, and they fall to the ground (or onto surfaces) in a few seconds. The spread of coronavirus and mortality at a location is influenced by many factors, including the health of the people, the personal and environmental opportunities for spread (e.g., the distance between people, temperature, availability of water and disinfectants), and the type of treatment and welfare schemes available. During the past year, several vaccines for COVID-19 were also developed and inoculated to millions of people around the world (as of 11 December 2021, a total of more than 8.41 billion vaccine doses have been administered around the world; World Health Organization 2021), with some notable success. However, despite our efforts and progress in dealing with COVID-19 (and earlier coronavirus-related and other infectious diseases), there are still enormous difficulties in understanding the spread of COVID-19 and controlling such spread. A key reason for these difficulties is the complex and nonlinear dynamic characteristics of each of the influencing factors (e.g., weather conditions, human hygiene, human movements and social activities) and also the interactions among them.

During the past few decades, various scientific approaches and mathematical methods were developed and applied to model the dynamics of the spread of infectious diseases [11,12,13,14,15,16,17,18,19,20]. Numerous studies have also examined the dynamics of SARS [21,22,23,24] and COVID-19 [25,26,27,28,29,30,31,32,33,34,35,36]. Many studies have specifically examined the nonlinear and chaotic dynamic nature of infectious diseases, including COVID-19 [12,13,15,19,37,38]. The outcomes of these chaos-based studies are certainly encouraging, as they indicate the possibility of developing fairly simple models to examine and predict the apparently complex and random-looking dynamics in the spread of infectious diseases, including COVID-19.

Motivated by such studies and outcomes, in the present study, we employ the concepts of nonlinear dynamic and chaos theories to examine the temporal dynamics of the spread of COVID-19 around the world. More specifically, we assess the complexity (or variability) of the dynamics of COVID-19 through estimating the dimensionality of COVID-19 data (i.e., COVID-19 cases and COVID-19 deaths). To this end, we apply, for the first time, the false nearest neighbor (FNN) method [39], a nonlinear dynamic dimensionality-based method, to examine the complexity of the COVID-19 time series. The complexity of the COVID-19 dynamics is represented by the FNN dimension, which is an indication of the number of variables dominantly governing the underlying system dynamics—The higher the dimensionality the greater the complexity, and the lower the dimensionality the lesser the complexity. The FNN method involves reconstruction of a single-variable time series in a multi-dimensional phase space to represent the underlying dynamics [40,41] and estimation of dimensionality using a nearest neighbor search in the reconstructed phase space [39].

To study the temporal dynamics of COVID-19, we consider two different types of COVID-19 time series from 40 countries/regions around the world. The two types of time series are: (1) daily COVID-19 cases; and (2) daily COVID-19 deaths. The data are obtained from the Our World in Data (OWID) website: https://ourworldindata.org/ (accessed on 30 March 2021). The 40 countries/regions considered in this study include 33 countries (including many of the countries that have been severely affected, such as Brazil, Germany, India, Italy, the United Kingdom, and the United States) and seven regions (Africa, Asia, Europe, European Union, North America, South America, and World). Since the start of COVID-19 happened at different times in different countries, the starting times of data available and, hence, used in this study for different countries/regions are also different, but data until 20 March 2021 are considered for all the countries/regions studied. However, for each country/region, at least one year of data is considered.

The rest of this paper is organized as follows. Section 2 presents some details of the COVID-19 data (i.e., cases and deaths) considered for analysis in this study and also describes the false nearest neighbor method. The results from the application of the FNN method to COVID-19 data are presented in Section 3, and a discussion of these results is made in Section 4. Important conclusions and scope for further research are reported in Section 5.

## 2. Materials and Methods

### 2.1. Study Area and Data

In the present study, to examine the temporal dynamics of COVID-19 spread, COVID-19 data from 40 countries/regions are studied. The COVID-19 data are obtained from the Our World in Data (OWID) website: https://ourworldindata.org/ (accessed on 30 March 2021). The OWID database provides daily COVID-19 data for all the countries and territories around the world. The database also provides data for different regions or continents, as appropriate. For instance, in addition to the countries and territories, data for Africa, Asia, Europe, European Union, International, North America, Oceania, South America, and World are also available. In addition to daily COVID-19 cases and COVID-19 deaths, the OWID data also provide some other statistics, such as COVID-19 cases per million, COVID-19 deaths per million, new tests, and many others. Since COVID-19 started to occur at different times in different countries/regions, the starting times of data available for different countries/regions are also different.

The 40 countries/regions considered in this study include 33 countries and seven regions, as listed in Table 1. They are selected primarily based on the following considerations: (1) at least 500,000 cases until 20 March 2021; (2) data are available for a reasonably long period (at least one year) and continuously from the starting date until 20 March 2021; (3) data are generally accurate (any perceived incorrect data are rectified as much as possible, through cross-verifying with other reliable data sources); and (4) global coverage, considering countries from all the major continents. The 33 countries include many of the countries that have been severely affected, such as Brazil, Germany, India, Italy, the United Kingdom, and the United States. The seven regions include Africa, Asia, Europe, European Union, North America, South America, and World; that is, except Oceania, all the major continents/regions are considered. It is appropriate to mention that a few countries that have also been significantly affected by COVID-19 are not considered for analysis here. Among such countries are China, Peru, Switzerland, and Sweden. These countries are not included because the data are either short or not very accurate (too many data seem uncertain).

In the present study, daily COVID-19 cases and daily COVID-19 deaths in the above 40 countries/regions are analyzed. Therefore, there are 40 time series of daily COVID-19 cases and 40 time series of daily COVID-19 deaths. Table 1 presents a summary of the COVID-19 cases and COVID-19 deaths in the 40 countries/regions, including the starting date of data available, total number of cases and deaths, as well as the mean and standard deviation of daily cases and daily deaths.

As can be realized from Table 1, there are significant differences in the statistics among the countries/regions—The cases and deaths when estimated out of a million (not presented in Table 1) in each of the 40 countries/regions also show significant differences. Further, the coefficient of variation (CV) (defined as the ratio of standard deviation to mean), which is a widely used statistic to measure the variability of a time series, also shows significant differences. For instance, the CV value for the daily COVID-19 cases ranges from 0.70 (for Bangladesh) to 1.62 (for Belgium) and that for the daily COVID-19 deaths ranges from 0.58 (for Bangladesh) to 1.44 (for Argentina); see Section 4 for additional discussion of the CV values, especially as a comparison with dimensionality.

**Figure 1:**
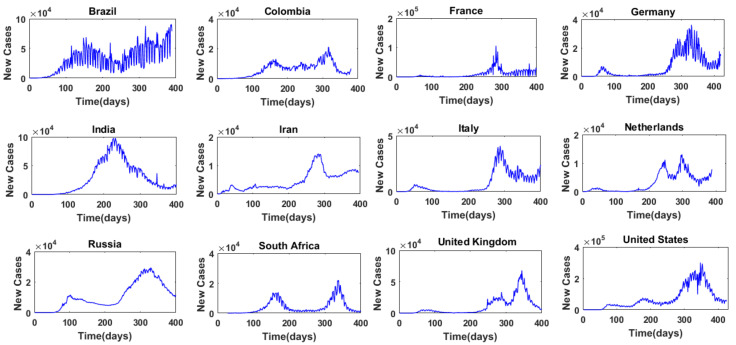
Time series of daily COVID-19 cases from 12 selected countries.

**Figure 2:**
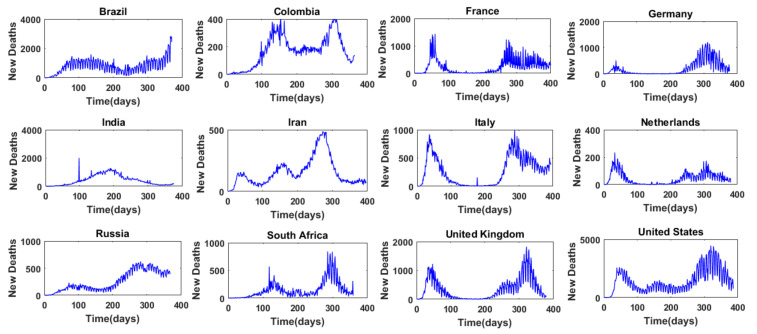
Time series of daily COVID-19 deaths from 12 selected countries.

Figure 1 presents, for example, the time series of daily COVID-19 cases in 12 selected countries, and Figure 2 presents the time series of daily COVID-19 deaths in these 12 countries. These 12 countries are: Brazil, Colombia, France, Germany, India, Iran, Italy, the Netherlands, Russia, South Africa, the United Kingdom, and the United States. They are selected for the purpose of illustration here, and detailed results are presented only for these 12 countries. It may be noted that these countries also cover almost all the major continents (Africa, Asia, Europe, North America, and South America). As can be seen from Figure 1 and Figure 2, there are some notable differences in the variations of the daily COVID-19 cases and daily COVID-19 deaths among the different countries. This seems to suggest that the selection of these 12 countries for the purpose of illustration of the complexity of COVID-19 cases and COVID-19 deaths around the world is reasonable.

### 2.2. Methodology—False Nearest Neighbor Algorithm

A popular way to assess the complexity of a dynamic system is through determining the dimensionality of a time series representing the system. The dimensionality of a time series is often considered a reliable indicator of the number of variables dominantly governing the underlying system dynamics. In the context of chaos theory, many dimension measures and methods were proposed. These include correlation dimension, capacity dimension, information dimension, Lyapunov dimension, and false nearest neighbor dimension; see, for example, [42] for details. Each of these dimension measures and methods has its own advantages and limitations. Therefore, a definitive conclusion as to which method is the best is hard to provide. However, the false nearest neighbor (FNN) method, originally proposed by [39], is often regarded as one of the better methods for dimension estimation, as it eliminates the “false” neighbors in the search for nearest neighbors in the reconstructed phase space (see below for details on phase space reconstruction). In addition, the method also works generally well for short and noisy time series. In view of these advantages, the FNN method [39] is employed in this study to estimate the dimensionality of the COVID-19 time series. A brief description of the FNN method is presented next.

The FNN method uses the concept of phase space to represent the evolution of the dynamics of a system, based on a time series representing the system. Phase space is essentially a graph or a coordinate diagram, with the coordinates representing the variables necessary to completely describe the state of the system at any moment [40]. The trajectories of the phase space diagram describe the evolution of the system from some initial state, which is assumed to be known and, hence, represents the history of the system. A point in the phase space represents the state of the system at any moment. Phase space can be reconstructed based on a single-variable time series (or multi-variable time series, if available). The idea behind such a reconstruction is that a (nonlinear) system is characterized by self-interaction so that a time series of a single variable (or multiple variables) can carry the information about the dynamics of the entire multi-variable system.

Many methods are available for phase space reconstruction from a single-variable time series [41,43,44,45]. Among these, the method of delay [41] is the most widely used one. According to this method, given a single-variable time series, *X_i_,* where *i* = 1, 2, …, *N*, a multi-dimensional phase space can be reconstructed as:

***Y****_j_= (X_j_*, *X_j+τ_*, *X_j+2τ_*, *…*, *X_j+(m–1)τ/∆t)_* (1)

where *j =* 1, 2, …, *N* − (*m* – 1)*τ*/*∆t*; *m* is the dimension of the vector ***Y****_j_*, called embedding dimension; and *τ* is an appropriate delay time taken to be a suitable integer multiple of the sampling time *∆t*. A correct phase space reconstruction in a dimension *m* generally allows interpretation of the system dynamics in the form of an *m*-dimensional map *f_T_*, given by:

***Y****_j+T_= f_T_*(***Y****_j_*) (2)

where ***Y****_j_* and ***Y****_j_*_+*T*_ are vectors of dimension *m*, describing the state of the system at times *j* (current state) and *j* + *T* (future state), respectively.

With the reconstruction of the phase space, the FNN algorithm can be described as follows. The algorithm examines, in dimension *m*, the nearest neighbor ***Y_j_****^NN^* of every vector ***Y****_j_*, as it behaves in dimension *m* + 1. If ***Y_j_****^NN^* is a true neighbor of ***Y****_j_*, then it comes to the neighborhood of ***Y****_j_* through dynamic origins. On the other hand, if ***Y****_j_^NN^* moves far away from ***Y****_j_* as the dimension is increased, then it is declared as a “false nearest neighbor”, as it arrived in the neighborhood of ***Y****_j_* in dimension *m* by projection from a distant part of the attractor. It is literally when moving to a dimension *m* + 1 from *m* in phase space, the number of false neighbors for each point of *m* + 1 goes to zero, whereas the number of false neighbors for each point is non-zero in dimension *m*.

To determine the optimum embedding dimension (*m_opt_*) while increasing the dimension to check if a nearest neighbor is false, loneliness criterion and distance criterion are generally used [46]. The loneliness criterion is used to check that the points reach a maximum stretch and cannot move further beyond when the dimension is increased, while the distance criterion is used to check whether a nearest neighbor moves far apart when the dimension is increased. The following tolerance guidelines are generally adopted for these:

Loneliness tolerance: If *R_m_*_+1_(*j*) ≥ 2*R_A_* (where *R_m_*_+1_(*j*) is the distance to the nearest neighbor of the *j*th vector (i.e., ***Y_j_****^NN^*) in an embedding of dimension (*m* + 1) and *R_A_* is the standard deviation of the time series *X_i_*), the *j*th vector has a false nearest neighbor.

Distance tolerance: If [*R_m_*_+1_(*j*) − *R_m_*(*j*)] > *ε R_m_*(*j*) (where *ε* is a threshold factor), the *j*th vector has a false nearest neighbor. The distance *R_m_*_+1_(*j*) is computed to the same neighbor that was identified with embedding *m*, but with the (*m* + 1)th coordinate (i.e., *X_j-mτ_* appended to the *j*th vector and to its nearest neighbor with embedding *m*). The appropriate threshold *ε* is generally selected through experimentation and is generally between 10 and 50 [46].

With the above conceptual description of the FNN algorithm, the specific steps involved in the implementation of the algorithm are as follows:

(i) Form the set of reconstructed vectors in the phase space using Equation (1) with embedding dimension *m*, say, *m* = 2;
(ii) Identify the nearest vector (in the Euclidean sense) for a given reconstructed vector in the phase space. That is, for the given reconstructed vector ***Y****j*, find the vector that has the minimum Euclidean distance with respect to ***Y****j*;
(iii) Check whether the loneliness tolerance criterion and the distance tolerance criterion are true or false. If both criteria are true, then the identified neighbor for the re-constructed vector ***Y****j* is false;
(iv) Continue the algorithm for the remaining reconstructed vectors. Calculate the total number of false nearest neighbors. The percentage of FNN (%FNN) is obtained by dividing the number of false nearest neighbors for embedding dimension *m* by the number of false nearest neighbors for embedding dimension 1;
(v) Perform the algorithm for increasing m until the percentage of false nearest neighbors drops to zero. The embedding dimension that yields zero or the lowest %FNN is then chosen as the optimal embedding dimension (*mopt*) or the “FNN dimension”.

Since the original FNN algorithm [39], several modifications were proposed in the literature; see, for example, [47,48,49,50,51] among others. While such modifications have yielded some improvements, they still possess certain limitations that are part of the original algorithm [39]. Many past studies have tested the reliability of the original algorithm [39] by applying it to several synthetic chaotic time series (logistic equation, Henon map, Lorenz map, Rössler map, Mackay-Glass equation), noise-added synthetic chaotic time series (with different levels of noise added to the clean chaotic series), and also stochastic time series (independent and identically distributed random time series, normally distributed random time series). It is also widely accepted, through comparison with other dimension estimation methods, that the original algorithm offers reliable results on the FNN dimension estimation. In view of these, we employ the original FNN algorithm [39] in the present study for estimating the dimension of the COVID-19 time series.

## 3. Analysis and Results

### 3.1. Analysis

The false nearest neighbor (FNN) algorithm is applied to each of the time series of the daily COVID-19 cases and daily COVID-19 deaths in the 40 countries/regions. For each time series, embedding dimension, m, values from 1 to 15 are considered for phase space reconstruction and subsequent FNN dimension analysis. The phase space reconstruction is performed with a delay time (*_τ_*) value of 1. Both the loneliness criterion and the distance criterion are considered to determine whether a nearest neighbor is false, and the following conditions are adopted: (1) *R_m_*_+1_(*j*) ≥ 2*R_A_*; and (2) threshold *ε* = 15. The embedding dimension that yields zero or the lowest percentage of false nearest neighbors (%FNN) is then chosen as the optimal embedding dimension or the “FNN dimension”. If more than one embedding dimension value gives the same %FNN, the lower (lowest) dimension is chosen as the FNN dimension. As mentioned earlier, for the purpose of illustration here, results for only 12 countries are presented in detail below. The time series of daily COVID-19 cases and daily COVID-19 deaths for these 12 countries have already been presented in Figure 1 and Figure 2, respectively.

### 3.2. Results for Daily COVID-19 Cases

**Figure 3:**
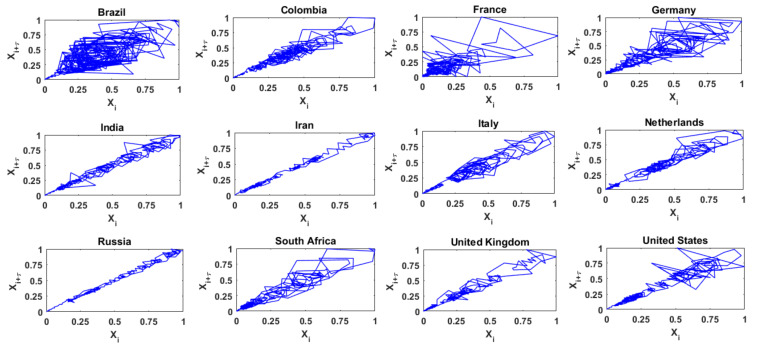
Phase space diagrams of daily COVID-19 cases from 12 selected countries.

Figure 3 presents the two-dimensional phase space diagrams (i.e., *m* = 2) for the time series of daily COVID-19 cases from the above 12 countries, with delay time (*_τ_*) = 1, i.e., the projection of the attractor on the plane {*X_i_*, *X_i_*_+1_}. In these diagrams, for each country, the values of COVID-19 cases are normalized to range from 0 to 1, for better visualization and comparison. The phase space diagrams for the 12 series exhibit certain noticeable differences. For instance, the trajectories for the time series of the daily COVID-19 cases from Brazil, France, and Germany occupy a large region in the reconstructed phase space; the trajectories for the series from India, Iran, Italy, Russia, and the United Kingdom occupy only a very narrow region; and the trajectories for the series from Colombia, the Netherlands, South Africa, and the United States lie between the above two. These observations seem to suggest that, considering the 12 countries, the time series of daily COVID-19 cases from the first group of countries above exhibit a relatively high level of complexity (or dimensionality), from the second group of countries exhibit a relatively low level of complexity, and the third group of countries exhibit an intermediate level of complexity. However, caution must be exercised in this kind of interpretation, since the phase space diagram is only a qualitative measure of complexity, and two-dimensional here. Therefore, a quantitative measure (such as dimensionality) would be more appropriate and necessary to measure the level of complexity, especially since the actual dimension of the time series may be much higher than the two dimensions.

**Figure 4:**
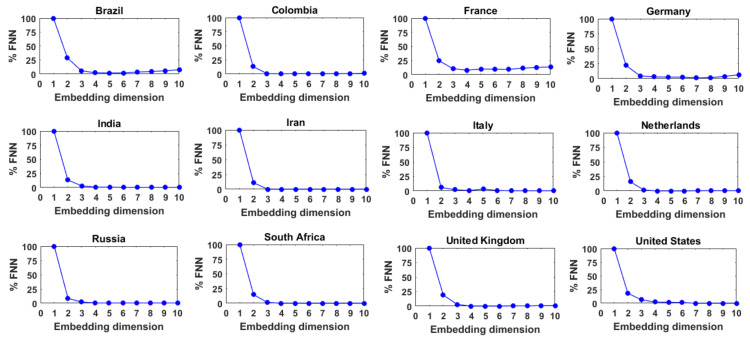
FNN dimension results for daily COVID-19 cases from 12 selected countries.

Figure 4 presents the results of the FNN analysis for these 12 series of daily COVID-19 cases, with *_τ_* = 1. As can be seen, for all the 12 series, the %FNN value generally decreases with an increase in the embedding dimension up to a certain point and then saturates or slowly increases after reaching the minimum. The optimum embedding dimensions or FNN dimensions (i.e., the embedding dimension that yields the lowest %FNN value) for these 12 series of daily COVID-19 cases are found to be 5, 4, 4, 7, 6, 3, 7, 4, 4, 4, 4, and 7, respectively. These low- to medium-level dimension values seem to suggest that the dynamics of the daily COVID-19 cases in these 12 countries exhibit low- to medium-level complexity. The dimension values also suggest that the daily COVID-19 cases in these 12 countries are dominantly governed by 3 to 7 variables, as appropriate for the individual countries.

**Figure 5:**
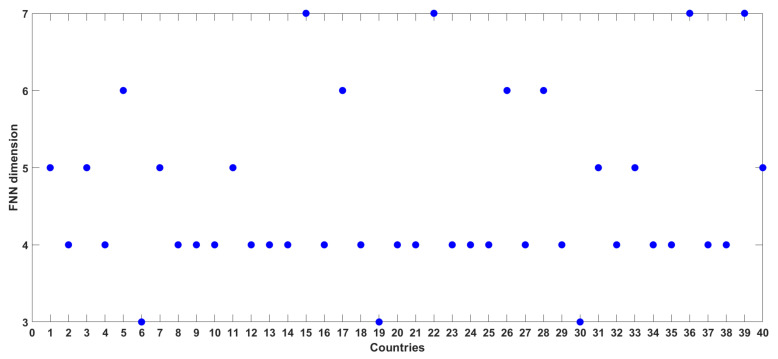
FNN dimension results for daily COVID-19 cases from 40 countries/regions.

Figure 5 presents the FNN dimension results for the time series of COVID-19 cases from all the 40 countries/regions considered in this study. The numbers (on the X-axis) in this figure correspond to the numbers for the countries/regions listed in Table 1. The FNN dimensions for the 40 series of COVID-19 cases range from 3 to 7, i.e., the same range for the 12 countries illustrated above. The break-down of the dimensions is as follows: Three countries (Belgium, Iran, and Portugal) have an FNN dimension of 3; 22 countries/regions (including Europe, European Union, and South America) have an FNN dimension of 4; seven countries/regions (including Africa, Asia, and World) have an FNN dimension of 5; four countries/regions (including North America) have an FNN dimension of 6; and four countries (Germany, Italy, Turkey, and the United States) have an FNN dimension of 7. Again, these low- to medium-level dimension values seem to suggest that the dynamics of the daily COVID-19 cases in the 40 countries exhibit low- to medium-level complexity and that they are dominantly governed by 3 to 7 variables, as appropriate for the individual countries/regions.

### 3.3. Results for Daily COVID-19 Deaths

**Figure 6:**
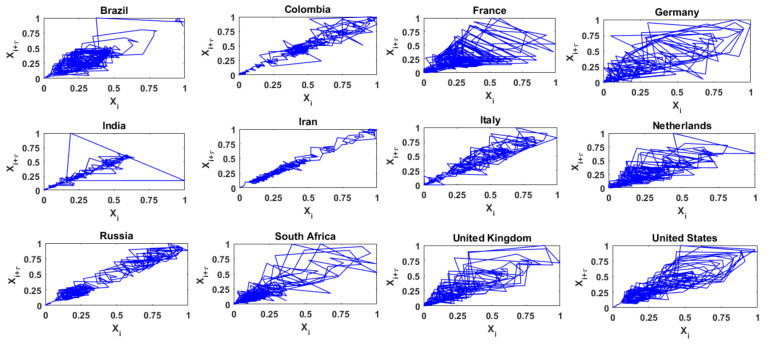
Phase space diagrams of daily COVID-19 deaths from 12 selected countries.

Figure 6 presents the two-dimensional phase space diagrams for the time series of daily COVID-19 deaths in the 12 countries mentioned above. As seen, the phase space diagrams for the 12 series of COVID-19 deaths exhibit significant differences among themselves. For most of the countries, the trajectories for the series of COVID-19 deaths occupy a large region in the reconstructed phase space, suggesting a relatively high level of complexity. The trajectories for the series of COVID-19 deaths from only Iran and Russia, and, to some extent, Colombia, Italy, and the United States occupy a relatively narrow region, suggesting a relatively low level of complexity. It may be noted that a comparison of these phase space diagrams for the daily COVID-19 deaths with those for the daily COVID-19 cases seems to indicate that the complexity of the former is greater than that of the latter (see Section 4 for additional details).

**Figure 7:**
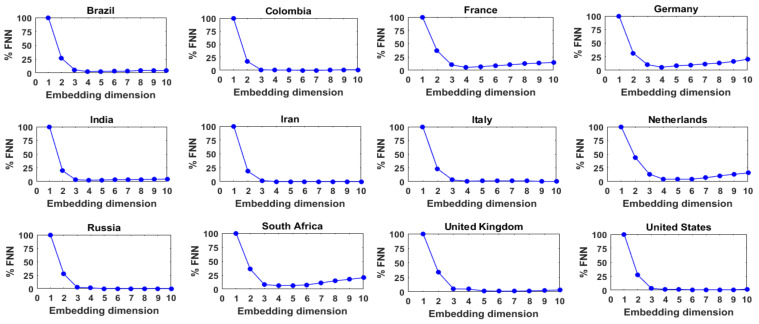
FNN dimension results for daily COVID-19 deaths from 12 selected countries.

Figure 7 presents the results of the FNN analysis for the time series of COVID-19 deaths from these 12 countries, with *τ* = 1. For all these 12 series, the %FNN value, in general, decreases with an increase in the embedding dimension up to a certain point and then saturates or slowly increases after reaching the minimum, similar to the ones observed for the COVID-19 cases. The FNN dimensions for these 12 series of COVID-19 deaths are found to be 4, 6, 4, 4, 4, 4, 9, 4, 5, 4, 6, and 6, respectively. Similar to the observations made for the daily COVID-19 cases above (Section 3.2), these low- to medium-level dimension values seem to suggest that the dynamics of the daily COVID-19 deaths in these 12 countries exhibit low- to medium-level complexity. The dimension values also suggest that the dynamics of the daily COVID-19 deaths in these 12 countries are dominantly governed by 3 to 9 variables, as appropriate for the individual countries.

**Figure 8:**
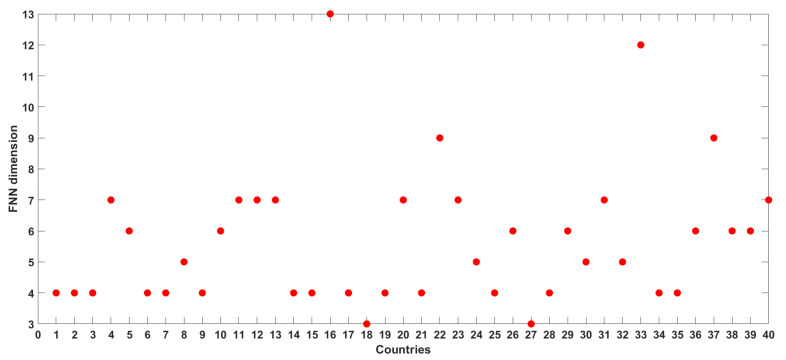
FNN dimension results for daily COVID-19 deaths from 40 countries/regions.

Figure 8 presents the FNN dimension results for the time series of COVID-19 deaths from all the 40 countries/regions. The FNN dimensions for these 40 series range from 3 to 13, and a more specific grouping is as follows. Two countries (Indonesia and Pakistan) have an FNN dimension of 3; 15 countries/regions (including Africa, Asia, and South America) have an FNN dimension of 4; four countries have an FNN dimension of 5; seven countries/regions (including North America) have an FNN dimension of 6; eight countries/regions (including Europe, European Union, and World) have an FNN dimension of 7; two countries (Italy and Ukraine) have an FNN dimension of 9; one country (Serbia) has an FNN dimension of 12; one country (Hungary) has an FNN dimension of 13. These dimension values, ranging from 3 to 13, seem to suggest that the dynamics of the daily COVID-19 deaths in the 40 countries exhibit anywhere from low to high level of complexity and that they are dominantly governed by 3 to 13 variables, as appropriate for the individual countries/regions.

## 4. Discussion

The FNN dimension results obtained in the present study for the time series of daily COVID-19 cases and daily COVID-19 deaths from 40 countries/regions may be discussed in different ways. These may include: (1) comparison of the dynamic complexity between COVID-19 cases and COVID-19 deaths; (2) nonlinear measure versus linear measure for assessing the variability or complexity of COVID-19 cases and COVID-19 deaths; and (3) data-related issues in the analysis of COVID-19 time series, especially considering the short and error-prone data.

### 4.1. Dynamic Complexity of COVID-19 Cases versus COVID-19 Deaths

**Figure 9:**
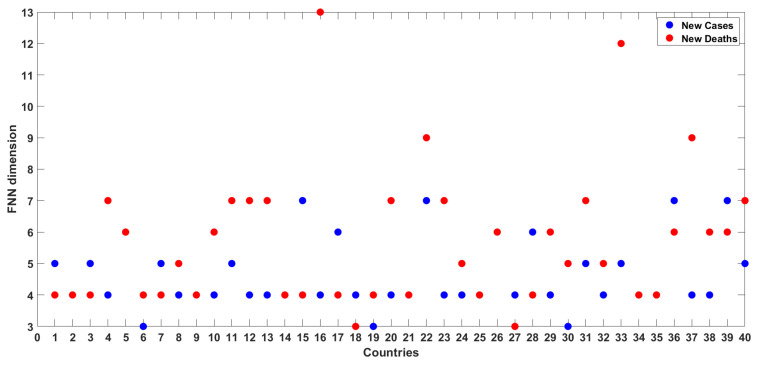
Comparison of FNN dimension results for daily COVID-19 cases and COVID-19 deaths from 40 countries/regions.

Figure 9 presents a comparison of the FNN dimensions obtained for the daily COVID-19 cases and daily COVID-19 deaths for all the 40 countries/regions considered in this study. As mentioned earlier, the dimensionality of the daily COVID-19 cases ranges from 3 to 7, suggesting low- to medium-level complexity, while the dimensionality of the daily COVID-19 deaths ranges from 3 to 13, suggesting low- to high-level complexity. Further, for a majority (three-fourth) of the countries/regions studied, the dimensionality of the daily COVID-19 deaths is greater than or at least equal to that of the daily COVID-19 cases. For instance, the dimensionality of the COVID-19 deaths is greater than that of the COVID-19 cases for 21 countries/regions, and the dimensionality is equal for COVID-19 cases and COVID-19 deaths for nine countries/regions. In addition, for a few countries/regions, the difference in the dimensionality between COVID-19 cases and COVID-19 deaths is significant (at least by a difference of 3), with greater dimensionality for the latter. This situation is especially observed for Hungary (dimensions are 13 and 4 for COVID-19 deaths and COVID-19 cases, respectively), Serbia (dimensions are 12 and 5), and Ukraine (dimensions are 9 and 4), and, to some extent, Austria, Europe, European Union, Iraq, and Jordan (dimensions are 7 and 4). However, the reverse situation, i.e., significantly greater dimensionality values for COVID-19 cases than that for COVID-19 deaths, is very rare, with the only exception of Germany (dimensions 7 and 4 for COVID-19 cases and COVID-19 deaths, respectively). These observations seem to indicate that, in general, the dynamics of daily COVID-19 deaths are more complex than that of COVID-19 cases. 

One contributing factor for the relatively less complexity in the dynamics of COVID-19 cases may be the number of COVID-19 tests conducted on a daily basis. It should be noted that, in many countries, there may not have been any significant difference in the number of tests conducted over a certain period (say, at least a few days) on a daily basis, which, in turn, could largely dictate the number of positive COVID-19 cases. Another contributing factor may be related to the extent of differences between the number of COVID-19 cases and COVID-19 deaths on a daily basis. For instance, the number of COVID-19 cases may have been increasing or decreasing at a relatively smaller level over a period of time on a daily basis, but, at the same time, there may be significant differences in the number of COVID-19 deaths over a period of time due to better treatments at some stages and also delays in the deaths during other periods.

### 4.2. Dimension versus Coefficient of Variation for COVID-19 Dynamics

As mentioned earlier, the FNN dimension is a representation of the variability and, hence, the complexity of the dynamics of a system in a nonlinear manner. One of the more useful statistical tools to assess the complexity of the dynamics of a system in a linear sense is the coefficient of variation (CV), which is the ratio of the standard deviation to the mean. With these, it would be useful to check whether the FNN dimension results are consistent with the CV values for the COVID-19 cases and COVID-19 deaths; in other words, higher FNN dimensions should have higher CV values, while lower FNN dimensions should have lower CV values.

**Figure 10:**
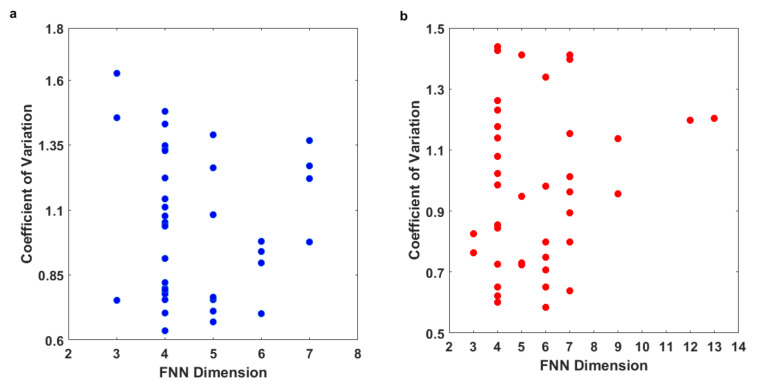
Relationship between FNN dimension and coefficient of variation (CV) for COVID-19 data from 40 countries/regions: (**a**) daily COVID-19 cases; and (**b**) daily COVID-19 deaths.

Table 2 presents the FNN dimensions and CV values for the daily COVID-19 cases and daily COVID-19 deaths from the 40 countries/regions considered in this study. Figure 10 presents the relationship between the FNN dimensions and the CV values for the COVID-19 cases (Figure 10a) and COVID-19 deaths (Figure 10b). The results do not seem to indicate any direct and consistent relationship between the FNN dimensions and the CV values, except for a very few countries/regions. For instance, for both COVID-19 cases and COVID-19 deaths, low and medium FNN dimension values (especially 3, 4, and 5 for COVID-19 cases and 4, 5, 6, and 7 for COVID-19 deaths) are observed for low, medium, and high CV values. However, high FNN dimension values (especially 7 for COVID-19 cases and 9, 12, and 13 for COVID-19 deaths) are observed mostly for medium and high CV values. This is particularly the situation for COVID-19 deaths (Figure 10b). As seen, for Hungary and Serbia, both the FNN dimensions (13 and 12) and the CV values (1.20 and 1.20) are relatively high.

These observations suggest that a direct relationship between the results from linear methods (e.g., CV) and those from nonlinear methods (e.g., FNN dimension) on the variability or complexity of a system may not always be possible to achieve. This interpretation is also consistent with that reported by several earlier studies that have applied the concepts of chaos theory, including the FNN method, in many other contexts [52,53].

### 4.3. Data-Related Issues for FNN Dimension Estimation

It is important to note that estimation of the FNN dimension of a time series may be influenced by data quantity and quality, such as data length and data error. Furthermore, the FNN dimension estimation may also be influenced by the parameters involved in phase space reconstruction, such as delay time (*τ*) and embedding dimension (*m*). Extensive details of the influence of these and several other data-related issues (e.g., presence of zeros, sampling frequency) on the FNN dimension estimation, and on chaos theory-based analysis of time series more broadly, can be seen in [46,47,50,51,54,55,56,57], among others. A brief account of the issues that are highly relevant to the COVID-19 data analyzed in this study is presented next.

**Data length**: In almost all time series analysis methods, it is preferable to have a longer time series (but at an appropriate scale) to achieve more reliable results. This is because, in general, a longer time series has a greater probability of covering and representing all the possible changes that a system undergoes. Several earlier studies on the application of chaos theory-based methods offered guidelines relating the data length to the attractor dimension or embedding dimension (especially in the correlation dimension estimation) and suggested the use of long time series to avoid potential underestimation of dimension; see [57] for details. However, Sivakumar [56] pointed out that the required data length often depends on whether the period of data is long enough to adequately represent the system dynamics (rather than the sheer number of values) and that reliable dimension estimates are possible even with short time series with about only 300 values. In view of this, we believe that the data used in this study for the 40 countries/regions (i.e., at least one year of daily data, amounting to 365 values) are long enough for an adequate representation of the COVID-19 dynamics and that the estimated dimensions are indeed reliable. This is particularly so, since the FNN algorithm generally works well for short (and noisy) time series [39]. Nevertheless, it is recommended to use even longer time series, to verify, and possibly confirm the present dimension results.

**Data quality**: The quality of data plays an important role in the reliability of the outcomes of any time series method, and the FNN method is no exception; see [55] for some details. For instance, the presence of errors (noise) in the data may often lead to an overestimation of the dimension. In the present study, we used the data from the Our World in Data (OWID) database. It is possible that this database contains some errors; indeed, we were able to identify some errors, in the context of the intended analysis. For instance, for a few countries, some days had zero COVID-19 cases and zero COVID-19 deaths, while the previous and subsequent days to such days had a high number of COVID-19 cases and COVID-19 deaths. We did not believe that this was the reality, but rather that the data were simply not reported for those (zero-recorded) days. In such cases, we also identified that the days subsequent to the days with zero cases/zero deaths had roughly double the number of cases/deaths reported for the days previous to them (i.e., simply the sum of two days of cases/deaths). Considering this issue and also the fact that the present study examines the daily COVID-19 cases and daily COVID-19 deaths, we made the necessary and appropriate corrections to the data, either by cross-checking with other information publicly available or by distributing the summed-up data over two days to the corresponding days. We believe that such corrections have helped in reducing the errors in the COVID-19 data used and resulted in more realistic data for use in the estimation of the dimension of the COVID-19 data at the daily scale. Having said that, examination of the influence of errors in the COVID-19 data on the FNN dimension estimates would also help to have even more confidence in the present outcomes. Such an examination may involve noise level determination and noise reduction. There exist many methods for noise level determination [58,59] and noise reduction [60,61]. Some studies have also proposed a coupled noise level determination–noise reduction approach for a more systematic analysis for chaos in a time series [62]. These methods can be applied to the COVID-19 data for more reliable outcomes.

**Delay time**: An appropriate delay time, *τ*, for phase space reconstruction is necessary to provide the best separation of neighboring trajectories within the minimum embedding phase space [63]. A too small or too large *τ* may lead to underestimation or overestimation of dimension. Many studies have addressed the issue of selection of *τ* and offered different guidelines, including the use of autocorrelation function [64],, average mutual information [54], and correlation integral [65]; see [57] for extensive details. However, different methods may result in different *τ* values for the same time series. Therefore, as of now, there is no definitive guideline on the selection of optimum *τ*. In this situation, some studies have adopted a trial-and-error procedure to examine the influence of *τ* on FNN (and any other) dimension estimation, by using multiple *τ* values [46,53]. Some other studies have used the delay time window, instead of the delay time [66,67]. In the present study, we used only one specific delay time value, i.e., *τ* = 1. We believe that using *τ* = 1 for the phase space reconstruction of the COVID-19 data is reasonable, especially considering that (1) it is the minimum possible *τ* value and provides daily separation of elements in the reconstruction; and (2) the length of the time series is rather short (365 values), and so increasing the value of *τ* can decrease the number of reconstructed vectors and constrain the neighbor search procedure in the FNN dimension estimation. However, examining the influence of *τ* on the FNN dimension estimation of the COVID-19 data would certainly help verify the reliability of the present results.

## 5. Conclusions

In the present study, we applied the chaos-theory-based false nearest neighbor (FNN) method to examine the complexity of the dynamics of COVID-19. We studied the daily COVID-19 cases and daily COVID-19 deaths in 40 countries/regions around the world. The results indicated that the COVID-19 cases exhibited low- to medium-level complexity (with dimensions in the range 3 to 7) and the COVID-19 deaths exhibited low- to high-level of complexity (with dimensions ranging from 3 to 13). For a majority (three-fourth) of the countries/regions studied, the complexity of the daily COVID-19 deaths was found to be greater than or at least equal to that of the daily COVID-19 cases.

The outcomes from this study have some important implications for modeling and prediction of the dynamics of COVID-19 (and other infectious diseases). The generally low- to medium-level complexity in the daily COVID-19 cases and daily COVID-19 deaths for the 40 different countries/regions suggest that the dynamics are not highly complex; only two countries (Hungary and Serbia) have a dimension larger than 10 (for COVID-19 deaths). Even for the countries/regions that have been very severely affected by COVID-19 at least in terms of the total population (e.g., the United States, Brazil, India, and the United Kingdom), the dimensionality of COVID-19 cases and COVID-19 deaths is fairly low, i.e., less than or equal to 7. This indicates that only a very few influencing factors dominate the spread of COVID-19 even in the most affected countries/regions. These observations suggest that highly complex models may not be required to model and predict the COVID-19 dynamics. This, however, does not mean that modeling the spread of COVID-19 around the world is fairly simple. It is important to note that different factors may dominate the spread of COVID-19 dynamics in different countries/regions, since the general health of the people, environmental and other conditions (e.g., weather, population density, transportation facilities), personal and collective hygiene and immunity, and availability of treatment facilities and other resources may be different in different countries/regions. Therefore, proper identification of the most dominant factors governing the COVID-19 dynamics in different countries/regions is critical to study the spread of COVID-19 in the individual countries and its impacts. This is a much-needed area of research, and we intend to study this in the near future.

Although the results from the present study are encouraging, especially from the viewpoint of the complexity of the model required for COVID-19 dynamics (i.e., number of variables/factors that dominantly influence the COVID-19 dynamics and the associated data requirements), it is important to note that the present study applied only one method, i.e., the FNN algorithm, to examine the complexity of the daily COVID-19 dynamics. Due to the potential limitations of any chaos theory-based method (just as in the case of any other time series analysis method), it may be necessary to apply still other methods that can help further verify, and possibly confirm the present results. To this end, we plan to employ additional chaos theory-based methods to examine the dimensionality and other relevant invariants of COVID-19 data. Furthermore, although the COVID-19 data are of reasonable length and quality, the use of data over a longer period and better quality (than that are available now) would certainly help to achieve more reliable results. With the spread of COVID-19 continuing in most of the countries/regions around the world (with new variants from time to time, including the variant Delta in recent months and the variant Omicron that has just been identified), there are expectations that the pandemic will last at least for a few more months, and perhaps even for another year or two. If this indeed would be the case, then there would be an even longer period of data (hopefully) with better quality for more rigorous analysis. We hope to re-visit the issues associated with COVID-19 data in our future research.

Finally, it is very important to recognize that despite the efforts made by various governments and organizations around the world (including those at global, national, and local levels) since the inception of COVID-19, there are still serious concerns about our ability to properly test COVID-19 on large populations at any given time, gather data on COVID-19 cases and COVID-19 deaths from the different locations, and compile and get the data readily available for use, both by governments/organizations and for academic/research purposes. Indeed, there remain concerns about the willingness of governments around the world to provide accurate data on COVID-19. These can have serious implications for our efforts to prevent, treat, and control the spread of COVID-19, reduce the loss of life, mitigate social and personal problems arising from COVID-19, and save our economy. For instance, low COVID-19 testing can result in the undercounting of cases and deaths in the majority of nations around the world, as has been reported by many studies [68,69,70,71]. Various factors can contribute to low COVID-19 testing, including economic resources, human resources, individual and societal health awareness, and political stability and will. In general, the undercounting of COVID-19 cases and deaths is highest in low-income countries. Properly addressing this undercounting of COVID-19 cases and deaths in countries/regions around the world, and other factors that influence the accuracy of COVID-19 data is critical to obtain reliable results and provide informed interpretations and conclusions from a study such as the present one (or any study, for that matter). We will look into this aspect in our future research.

## Figures and Tables

**Table 1:** Basic statistics of COVID-19 data from 40 countries/regions (End date: 20 March 2021).

S. No.	Country/Region	Starting Date	Cases			Deaths		
			Total	Daily Mean	Daily Std. Dev.	Total	Daily Mean	Daily Std. Dev.
			(×10^5^)					
1	Africa	13 February 2020	40.97	10,192	7706	109,674	272.8	230.3
2	Argentina	1 January 2020	22.5	5875	4615	54,595	144.4	207.9
3	Asia	22 January 2020	260.32	61,396	41,137	415,596	980.2	589.5
4	Austria	25 February 2020	5.11	1311	1741	9056	24.2	34.2
5	Bangladesh	3 March 2020	5.69	1484	1041	8668	23.6	13.8
6	Belgium	2 April 2020	8.36	2035	3306	22,268	59.5	73.2
7	Brazil	26 February 2020	121.15	31,143	22,131	292,742	793.3	492.4
8	Canada	26 January 2020	9.36	2228	2401	22,635	60	56.9
9	Chile	23 February 2020	9.01	2298	1615	22,180	60.9	76.9
10	Colombia	3 June 2020	23.31	6134	4628	61,907	170.1	110.6
11	Czechia	2 February 2020	14.59	3800	4792	24,735	68	78.5
12	Europe	23 January 2020	375.9	88,865	92,386	880,187	2082	2003
13	European Union	23 January 2020	249.81	59,055	65,546	592,818	1401	1412
14	France	24 January 2020	43.58	10,326	13,774	93,092	232.7	273.7
15	Germany	27 January 2020	27.44	6548	8306	73,740	195.6	278.9
16	Hungary	3 April 2020	5.61	1468	2100	18,068	48.7	58.6
17	India	30 January 2020	115.74	27,889	27,319	160,230	427.3	365.4
18	Indonesia	3 February 2020	14.56	3791	3459	39,447	105.2	80.2
19	Iran	19 February 2020	17.94	4529	3411	61,724	155.9	113.2
20	Iraq	24 February 2020	7.89	2018	1613	13,969	36.6	32.7
21	Israel	20 February 2020	8.31	2108	2410	6116	16.7	16.5
22	Italy	31 January 2020	33.57	8088	9868	104,704	265.7	254.2
23	Jordan	3 March 2020	5.27	1375	2036	5788	16.1	22.5
24	Mexico	1 January 2020	21.85	5645	4389	197,141	537.2	391.5
25	Netherlands	2 September 2020	12.12	3122	3288	16,473	43.4	44.3
26	North America	22 January 2020	343.16	80,933	76,104	789,790	1862	1395
27	Pakistan	25 February 2020	6.38	1640	1345	13,971	38	31.4
28	Philippines	30 January 2020	6.56	1577	1411	12,934	31.3	35.7
29	Poland	3 April 2020	20.37	5331	7179	49,159	131.4	176.1
30	Portugal	3 January 2020	8.17	2128	3096	16,762	45.4	64.1
31	Romania	26 February 2020	8.93	2295	2481	22,132	60.8	48.6
32	Russia	31 January 2020	43.98	10,597	8425	93,090	253.7	183.8
33	Serbia	26 February 2020	5.49	1444	2007	4906	13.4	16.1
34	South Africa	2 July 2020	15.37	3766	4606	52,082	145.1	156.6
35	South America	22 February 2020	199.17	50,679	32,165	516,083	1313	854.3
36	Turkey	3 November 2020	21.99	5866	8021	29,959	81.2	64.9
37	Ukraine	3 March 2020	43.05	10,373	13,770	126,359	332.5	378.2
38	United Kingdom	31 January 2020	15.85	4138	4294	31,352	84.1	82.5
39	United States	22 January 2020	297.83	70,242	68,664	541,914	1404	993.6
40	World	22 January 2020	1219.8	287,706	220,234	2,709,610	6391	4074

**Table 2:** FNN dimension values and coefficient of variation values for daily COVID-19 cases and COVID-19 deaths from 40 countries/regions.

S. No.	Country	Cases		Deaths	
		FNN Dimension	CV	FNN Dimension	CV
1	Africa	5	0.76	4	0.84
2	Argentina	4	0.79	4	1.44
3	Asia	5	0.67	4	0.60
4	Austria	4	1.33	7	1.41
5	Bangladesh	6	0.70	6	0.58
6	Belgium	3	1.62	4	1.23
7	Brazil	5	0.71	4	0.62
8	Canada	4	1.08	5	0.95
9	Chile	4	0.7	4	1.26
10	Colombia	4	0.75	6	0.65
11	Czechia	5	1.26	7	1.15
12	Europe	4	1.04	7	0.96
13	European Union	4	1.11	7	1.01
14	France	4	1.33	4	1.18
15	Germany	7	1.27	4	1.43
16	Hungary	4	1.43	13	1.20
17	India	6	0.98	4	0.86
18	Indonesia	4	0.91	3	0.76
19	Iran	3	0.75	4	0.73
20	Iraq	4	0.80	7	0.89
21	Israel	4	1.14	4	0.99
22	Italy	7	1.22	9	0.96
23	Jordan	4	1.48	7	1.40
24	Mexico	4	0.78	5	0.73
25	Netherlands	4	1.05	4	1.02
26	North America	6	0.94	6	0.75
27	Pakistan	4	0.82	3	0.83
28	Philippines	6	0.9	4	1.14
29	Poland	4	1.35	6	1.34
30	Portugal	3	1.45	5	1.41
31	Romania	5	1.08	7	0.8
32	Russia	4	0.80	5	0.72
33	Serbia	5	1.39	12	1.20
34	South Africa	4	1.22	4	1.08
35	South America	4	0.63	4	0.65
36	Turkey	7	1.37	6	0.80
37	Ukraine	4	1.33	9	1.14
38	United Kingdom	4	1.04	6	0.98
39	United States	7	0.98	6	0.71
40	World	5	0.77	7	0.64

## Data Availability

The COVID-19 data used in this study were downloaded from the One World in Data (OWID) database available at: https://ourworldindata.org/ (accessed on 30 March 2021). The data may be obtained from the authors upon request.

## Availability of Code

The code may be obtained from the authors upon request.
